# Unlocked Concanavalin A Forms Amyloid-like Fibrils from Coagulation of Long-lived “Crinkled” Intermediates

**DOI:** 10.1371/journal.pone.0068912

**Published:** 2013-07-16

**Authors:** Valeria Vetri, Maurizio Leone, Ludmilla A. Morozova-Roche, Bente Vestergaard, Vito Foderà

**Affiliations:** 1 Dipartimento di Fisica e Chimica, Università di Palermo, Palermo, Italy; 2 Department of Medical Biochemistry and Biophysics, Umeå University, Umeå, Sweden; 3 Department of Drug Design and Pharmacology, University of Copenhagen, Copenhagen, Denmark; 4 Sector of Biological and Soft Systems, Department of Physics, Cavendish Laboratory, University of Cambridge, Cambridge, United Kingdom; Russian Academy of Sciences, Institute for Biological Instrumentation, Russian Federation

## Abstract

Understanding the early events during amyloid aggregation processes is crucial to single out the involved molecular mechanisms and for designing *ad hoc* strategies to prevent and reverse amyloidogenic disorders. Here, we show that, in conditions in which the protein is positively charged and its conformational flexibility is enhanced, Concanavalin A leads to fibril formation *via* a non-conventional aggregation pathway. Using a combination of light scattering, circular dichroism, small angle X-ray scattering, intrinsic (Tryptophan) and extrinsic (ANS) fluorescence and confocal and 2-photon fluorescence microscopy we characterize the aggregation process as a function of the temperature. We highlight a multi-step pathway with the formation of an on-pathway long-lived intermediate and a subsequent coagulation of such “crinkled” precursors into amyloid-like fibrils. The process results in a temperature-dependent aggregation-coagulation pathway, with the late phase of coagulation determined by the interplay between hydrophobic and electrostatic forces. Our data provide evidence for the complex aggregation pathway for a protein with a highly flexible native conformation. We demonstrate the possibility to generate a long-lived intermediate whose proportion and occurrence are easily tunable by experimental parameters (i.e. temperature). As a consequence, in the case of aggregation processes developing through well-defined energy barriers, our results can open the way to new strategies to induce more stable *in vitro* on-pathway intermediate species through a minute change in the initial conformational flexibility of the protein. This will allow isolating and experimentally studying such transient species, often indicated as relevant in neurodegenerative diseases, both in terms of structural and cytotoxic properties.

## Introduction

Clarifying the multi-step process leading a native protein to lose its native structure and convert into amyloid aggregate is one of the most fascinating goals in biophysics. One reason is due to the still unclear connection between the amyloid deposits found in human tissues and the onset of devastating neurodegenerative pathologies as Alzheimeŕs and Parkinsońs diseases [1], [2]. Such deposits are mainly formed by elongated structures, known in the literature as amyloid fibrils. They are characterized by a common cross-β structure and they can present different isoforms potentially leading to different biological effects [3].

*In vitro* protein aggregation of model systems has provided significant insights on the various steps that lead to the formation of amyloid fibrils. However, a number of evidences have recently suggested that the formation of such elongated structures is only one of the possible amyloid-relevant pathways. As an example, very complex structures have been observed in large areas of Alzheimeŕs brains [4] and clustering of amyloid non-elongated material is often detected *in vivo* [5]. Moreover, such multiplicity of structures is also observed *in vitro*. In fact, under certain experimental conditions, a number of model systems (such as insulin, lysozyme and glucagon) can convert into amyloid aggregates with different shapes and characteristic sizes, varying from hundreds of nm to several µm. It has been reported that the formation of a particular amyloid structure strongly depends on the pH of the solution, salt concentration, protein concentration and shear stress [6]–[12]. Moreover, the balance between different interactions, regulated by multiple energy barriers, may give rise to differences in structure, morphology and toxicity of both fibrils and intermediate species [3], [13]–[15]. Depending on external conditions, protein assembly may proceed via different conformational changes, which drive the aggregation pathway toward a variety of polymorphic structures [8], [16]. Notwithstanding that the presence of such variety of structures is widely observed, its origin is still unclear. Particularly elusive are the early structural events in fibrillation, and how they can drive the formation of specific final arrangements.

Concanavalin A (Con A) is an all-β protein constituted by 237 amino acid residues [17]. Con A is extensively used as a model system for its ability to induce liver injury and for studying protein–carbohydrate interactions [18]–[19]. Characterizing the aggregation processes of Con A is of interest for a range of scientific applications. Con A agglutinates several eukaryotic cell-types [20], and shows characteristic binding specificity for bacterial surface-exposed carbohydrates [21]. Also, Con A has significant structural homology to the human serum amyloid protein (SAP) that is present in *in vivo* amyloid deposits [22]. Moreover, the absence of disulphide bonds in its structure enhances its flexibility, favoring conformational changes at the tertiary structure level [23]. Finally, the aggregation process of Con A is also associated with its antitumor activity [24], [25] and was found to be related to its property of inducing apoptosis on tumoral cells [26], [27]. It has been shown that at basic pH (away from the pI) well defined long and thin amyloid-like fibrils are formed and in parallel the native tetramer structure undergoes large conformational changes [28], [29]. This reaction proceeds via a non-nucleated assembly mechanism involving the formation of intermolecular β-sheets [28], [29]. Bringing the pH closer to the pI of the protein (i.e. pH 5.1) the process leads to the formation of amorphous aggregates [28]. These evidences suggest that the average charge per molecule may affect the specific aggregation pathway leading to a specific morphology at least for pH >pI. In this pH range, Con A is bound to Mn^2+^ and Ca^2+^ that provide stabilization of a polypeptide loop (residues 11–23) [30].

Here, we focus on the aggregation process of Con A under acidic conditions (pH 3). At low pH, away from pI, Con A adopts a less ordered structure, potentially combined to the dissociation of metals and loss of sugar binding capability. This can lead to what is called the “unlocked” structure with a consequent high conformational flexibility of the protein [30]. In these experimental conditions we can relate the combined effect of an enhanced conformational flexibility of the protein and the varied boundary conditions (i.e. overall positive charge, long range electrostatic forces and hydrophobic interactions) to both the aggregation kinetics and the species occurring along the process. Using circular dichroism, tryptophan (Trp) and ANS fluorescence, light scattering, confocal and two-photon excitation microscopy and small angle X-ray scattering, we analyze the secondary and tertiary conformational changes in relation to the final aggregate structure and morphology, highlighting a two-step temperature-dependent pathway that includes the coagulation of early on-pathway intermediates into amyloid-like elongated fibrils. By varying temperature, we influence the equilibrium between intermediate and late stage aggregates, and hence enable the detection of the intermediate on-pathway structures.

## Results and Discussion

### Con A Aggregation and Temperature Effects

**Figure 1:**
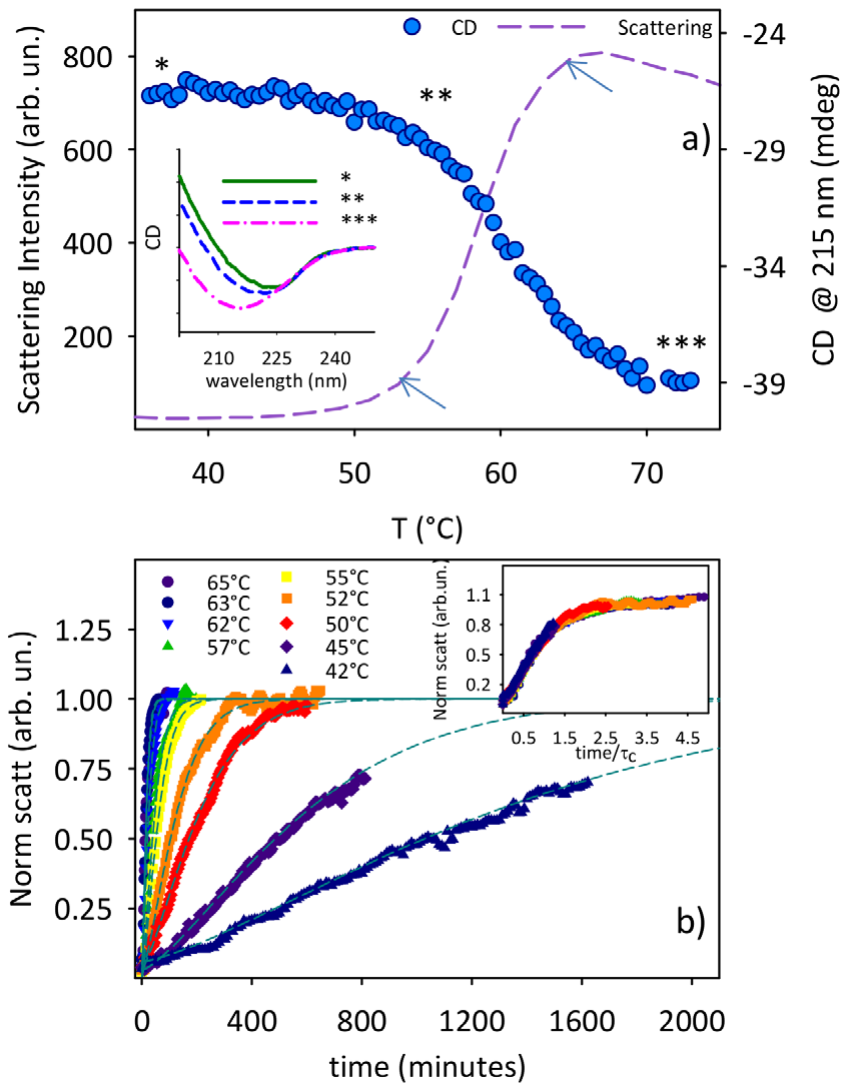
Effect of temperature on the aggregation process of unlocked Con A. (a) Elastic scattering intensity (λ = 633 nm) in back scattering configuration at 173°, measured during an upward temperature scan (12.5°C/h) for a 20 µM Con A sample at pH 3.0 (left axis), and CD signal measured at 215 nm in the same conditions (right axis). Arrows indicate regions of critical temperatures (changing of slope). The inset shows CD Spectra for three samples brought back at room temperature after upward temperature scan to the temperatures indicated by the asterisks (b) Kinetics of scattering intensity for 20 µM Con A samples incubated at 9 different temperatures ranging from 42 to 65°C. For each temperature, data were fitted using a compressed exponential function *y = A[1−exp(− (t/τ_c_)^b^)]*, with b = 1.4. Inset: kinetics of panel (b) normalized for *τ_c_* (x axis) and *A* (y axis).

In Figure 1a we report the elastic scattering intensity measured during an upward temperature scan for 20 µM Con A sample at pH 3. This approach is widely used to single out the critical temperatures at which the aggregation process is activated [23]. In our system, the scattering intensity remains unchanged up to ∼50°C and then a steep increase takes place, indicating that protein molecules lose their stability undergoing aggregation. In the proximity of this temperature, the protein molecule is partially destabilized. The protein structure is best considered as an ensemble of partially unfolded structures, exposing hydrophobic parts of the structure interior, creating conditions favorable to the formation of aggregates. Variation in external parameters, such as temperature, will shift the equilibrium, and hence influence the probability of aggregation. Interestingly, above 60°C a further change of slope in the profile is observed suggesting that the overall aggregation phenomenon could not be a simple two-state transition. Moreover, to monitor the effect of the increasing temperature on the secondary structure we measured changes in Far-UV Circular Dichroism (CD) spectrum. The CD signal was measured at 215 nm (Figure 1a) as a function of temperature during an upward temperature scan in the same conditions as the one used for scattering measurements. Changes in CD spectrum as a function of temperature stem for changes in protein secondary structure. The growth of the negative signal at about 215 nm was previously related to an enhanced β-aggregates content (i.e. not β-native) occurring in parallel with the supra-molecular assembly during the aggregation process of Con A samples at pH 9 [28], [29]. The inset shows the CD spectra of three homologous samples after three different upward temperature scans in the same condition. Scans were stopped at 37°C, 54° and 71°C (asterisks in the main panel). As can be seen, the shape of the spectrum undergoes significant changes whose major extent is at about 215 nm. The intensity of the CD signal at 215 nm shows a similar behavior as the one obtained for the scattering signal during the upward temperature scan (circles in Figure 1a): below 50°C weak changes are observed while at higher temperature the CD value decreases and above 65°C the signal reaches a plateau. Data in Figure 1a hence in concert suggest that at lower temperature the onset of the aggregation proceeds in parallel with changes in protein secondary structure. Both supra-molecular assembly and secondary structure changes present two critical temperatures slightly above T∼50°C and T∼60°C. Such evidence suggests that both secondary structure changes and supra-molecular aggregation have similar activation energy barriers. At temperatures higher than 60°C supra-molecular changes are further observed whilst changes in secondary structure seem to be completed at temperature higher than 65°C.

The analysis of the temperature dependence of the aggregation kinetics confirms that the system follows a transition more complex than a simple two-state transition. In Figure 1b we report the evolution of scattering intensity over time for the same sample measured at nine different temperatures ranging from 42°C to 65°C. All the kinetic profiles in Figure 1b present an increase in the scattering signal already in the very early stages of the process without any significant lag phase. We note that it is not surprising that aggregation processes are observed even below 50°C for long incubation times. At lower temperatures, the ensemble of structures is shifted more towards folded states, hence the probability of aggregation is lower, resulting in slower kinetics, yet the small fraction of fibrillation-prone (partially unfolded) proteins present will eventually result in the formation of aggregates [31]. The curves in Figure 1b cannot be described by a simple exponential growth while a *compressed* exponential function (i.e. *y = A [1−exp(− (t/τ_c_)^b^)] with b>1*) is more suitable at all the different temperatures (dotted lines in Figure 1b). The use of a compressed exponential law is not based on any particular model for supra-molecular assembly. However, it may indicate that the observed aggregation process involves different phases whose reaction rates change as a function of time [32], [33]. Possible scenarios could be determined by changes in the reactivity of the growing species in solution.

For each kinetic profile in Figure 1b, the *b* parameter assumes the same value (*b = 1.4*) thus indicating that at all temperatures the temporal evolution of the process follows the same law, with different rates 1/τ_c._ The similarity of the kinetic profiles can also be appreciated after the superimposition of the curves normalized by the parameter *A* (y axis) and *τ_c_* (x axis) (Figure 1b, inset). Such evidence suggests that at each temperature supra-molecular aggregation with similar features takes place. A pronounced temperature dependence is however evident and affects the temporal features of the kinetics with the growth of macromolecular aggregates being faster at higher temperatures (Figure 1b). All this is in accordance with a shift in the distribution of the structural ensemble towards a larger fraction of partially destabilized protein structures eventually taking part to the supra-molecular aggregation.

The aggregation kinetics in Figure 1b can be also fitted using a double exponential function. This would suggest two main steps of the process with different rates. This could be interpreted as a faster diffusion-limited initial aggregate formation and a slower reaction-limited supra-molecular assembly, the latter due to coagulation of aggregates formed in the earlier phase. However, this fitting would require at least 5 free input parameters in a global fit, making such kind of analysis unreliable. A similar kinetic behavior was found for knotted protein folding routes thus highlighting the complex folding pathway with intermediate states [32]. The analysis using the compressed exponential in Figure 1b puts in evidence the complexity of the observed aggregation process with a potential interplay of intermediate steps. However, at this stage we have no elements to univocally single out both the specific number of steps along the pathway and their features.

**Figure 2:**
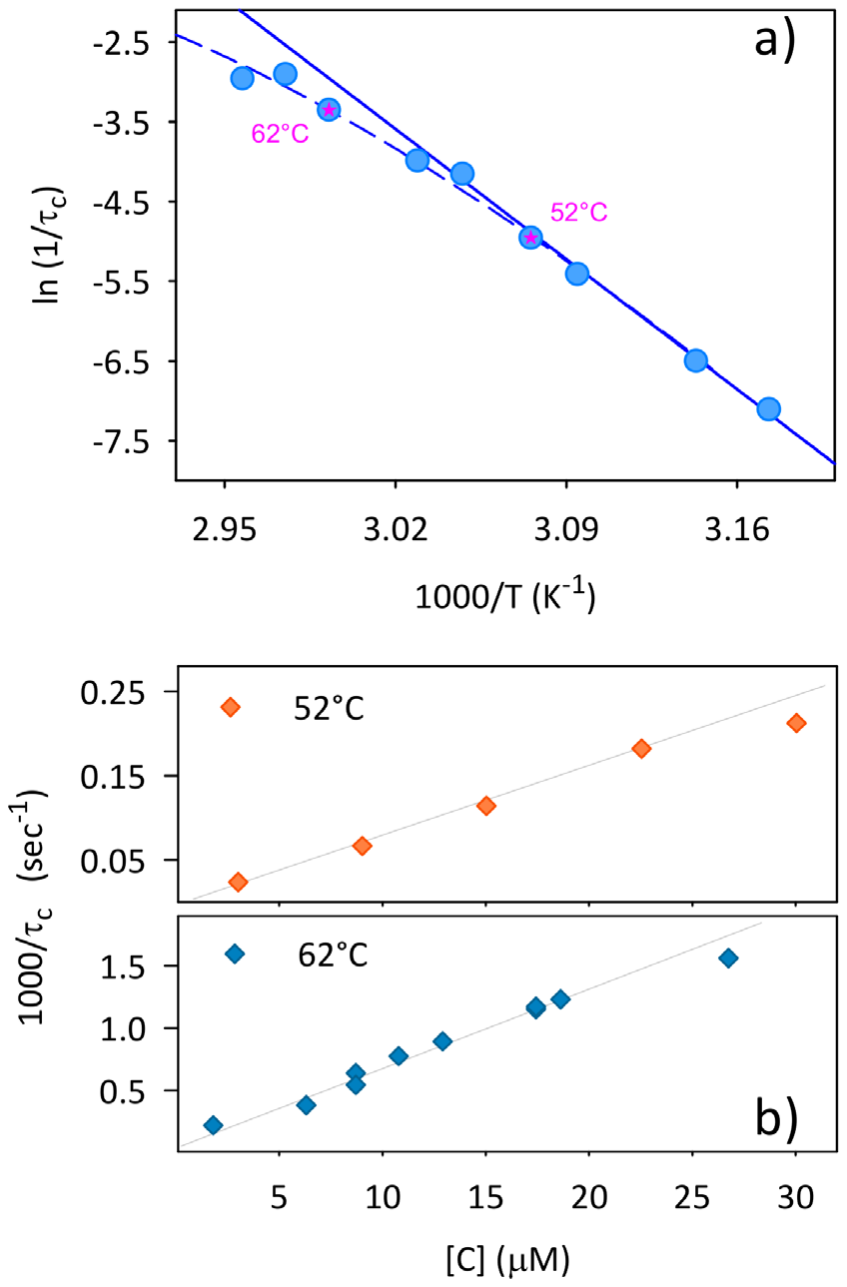
Deviations from Arrhenius behavior. (a) Arrhenius plot relative to data in Figure 1b. Solid line indicates the linear fit of the data in the range 42–57°C. Dashed line is a guide for eyes. (b) 1/*τ_c_* as a function of Con A concentration for the aggregation processes at 52 and 62°C.

Fitting the data in Figure 1b by means of a compressed exponential function (using only 2 free parameters, i.e. *A* and *τ_c_*, with a fixed *b = 1.4*) allows heuristically evaluating the rates (1/τ_c_) of the supra-molecular assembly. The rates are reported in the Arrhenius plot in Figure 2a. Up to 57°C estimated rates show a linear trend (solid line) with average activation energy of ∼200 kJ/mol, while at higher temperatures a deviation from linearity is observed. Importantly such a deviation is observed for temperature higher than 60°C, i.e. approximately in the same range as the second change of slope in the temperature scan (Figure 1a). Deviations from Arrhenius behavior can be attributed to differences in heat capacity between the ground state and transition state of the reaction and can generate multiple steps in the aggregation process depending on temperature [34]. We wish to note that heat capacity changes are generally interpreted as the change in exposed surface area accompanying the kinetic transition [34]. Aggregating proteins probably undergo structural changes leading the exposure to reactive areas, which concur in changing the aggregation pathway [34], [35]. Hence, the deviations from the Arrhenius behavior further strengthen the initial notion, based on temperature scans (Figures 1a and 1b), that the observed process is far from being a simple two-state transition. Specifically, this allows us to infer that aggregate formation evolves through at least two phases. During these phases, the system can explore different minima in an energy landscape characterized by temperature-dependent energy barriers with the process determined by the interplay and balance between different mechanisms.

An *ex situ* Thioflavin T (ThT) assay [36]–[38] was also performed on the final aggregates. At each temperature a weak ThT fluorescence signal was found, with a slightly higher intensity for aggregates obtained at higher temperatures (data not shown). However, the observed ThT intensity is 50-fold lower compared to the one previously obtained from large Con A fibrils formed at basic pH [28].

Data in Figure 2a suggest the presence of two distinct temperature ranges roughly identified below and above 57°C, in which the aggregation pathway could present kinetically different steps. To further investigate this, we selected two temperatures within these ranges. Hence, characterization of the aggregation process was performed at 52°C (slightly above the lowest critical temperature, Figure 1a and within the region of linearity of Figure 2a) and at 62°C, i.e. within the region of deviation from the Arrhenius behavior (Figure 2a). At both temperatures the aggregation process was monitored as a function of initial protein concentration in the range 3–30 µM. The scattering profiles resemble the one observed in Figure 1b and results are very similar at both temperatures and at each concentration. Also, in all cases no significant lag phase is detectable (data not shown). In Figure 2b the rate 1/τ_c_ is shown as a function of the protein concentration for the two temperatures. In both cases a linear trend is obtained. An aggregation rate linearly dependent on protein concentration suggests that supra-molecular association is not regulated by homogeneous nucleation mechanisms, in line with the observation that no significant lag phase is detected [39], [40]. However, this result does not exclude the presence of stochastic nucleation mechanisms [41] or high order reactions like coagulation processes, whose complexity and heterogeneity may “hide” the real concentration dependence [42]. Based on the above evidences and with the aim of clarifying the potential multi-step process highlighted by data in Figure 1, we focused on aggregation kinetics for samples at 20 µM Con A incubated at 52°C and 62°C.

### Secondary Structure Changes and Final Morphologies

**Figure 3:**
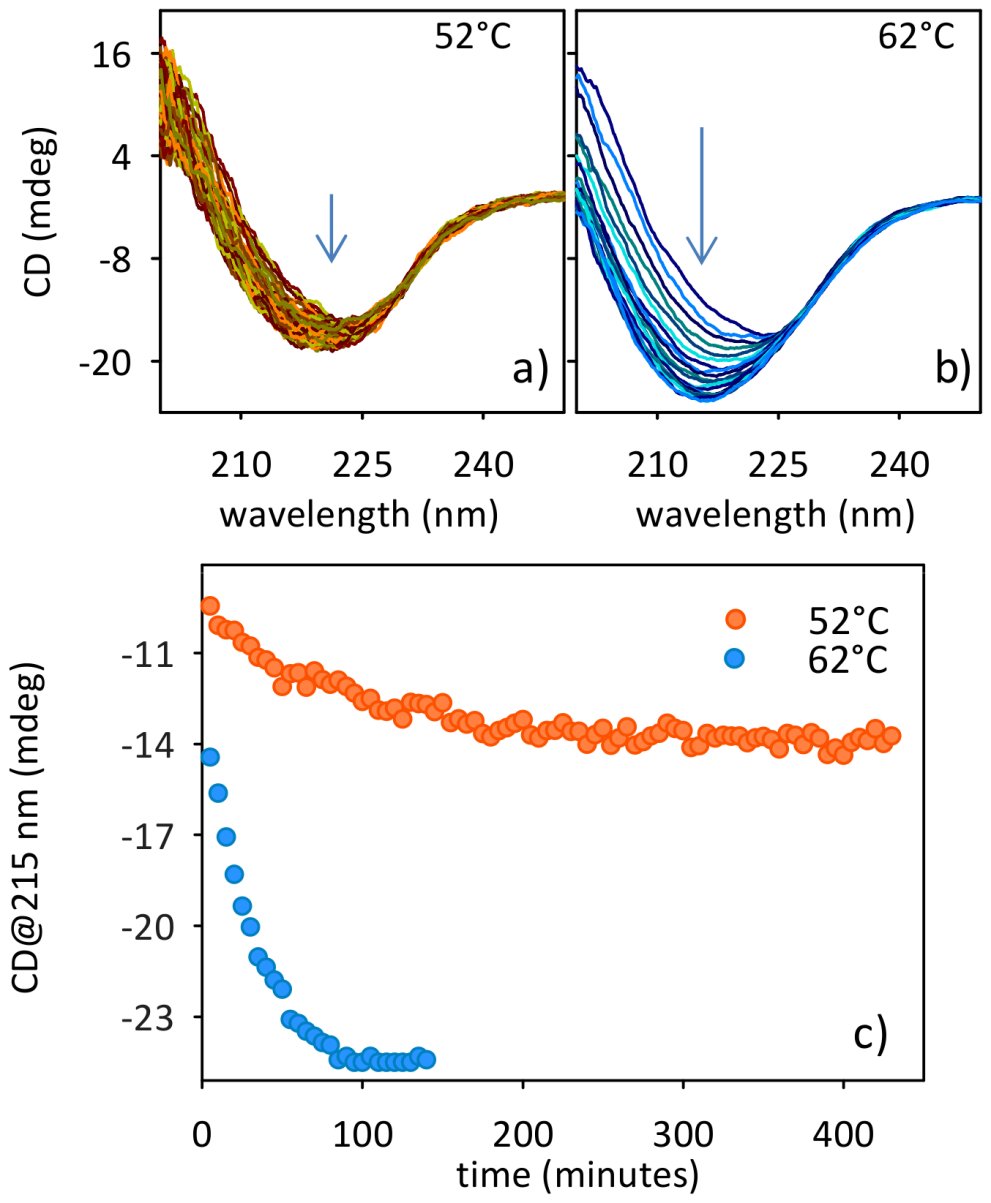
Secondary structure changes during the aggregation process. Circular dichroism spectra during the aggregation of 20 µM Con A sample at pH 3 at (a) 52°C and (b) 62°C. Arrows indicate spectra development as a function of time (c) CD signal at 215 nm during the kinetics at 52°C and 62°C.

**Figure 4:**
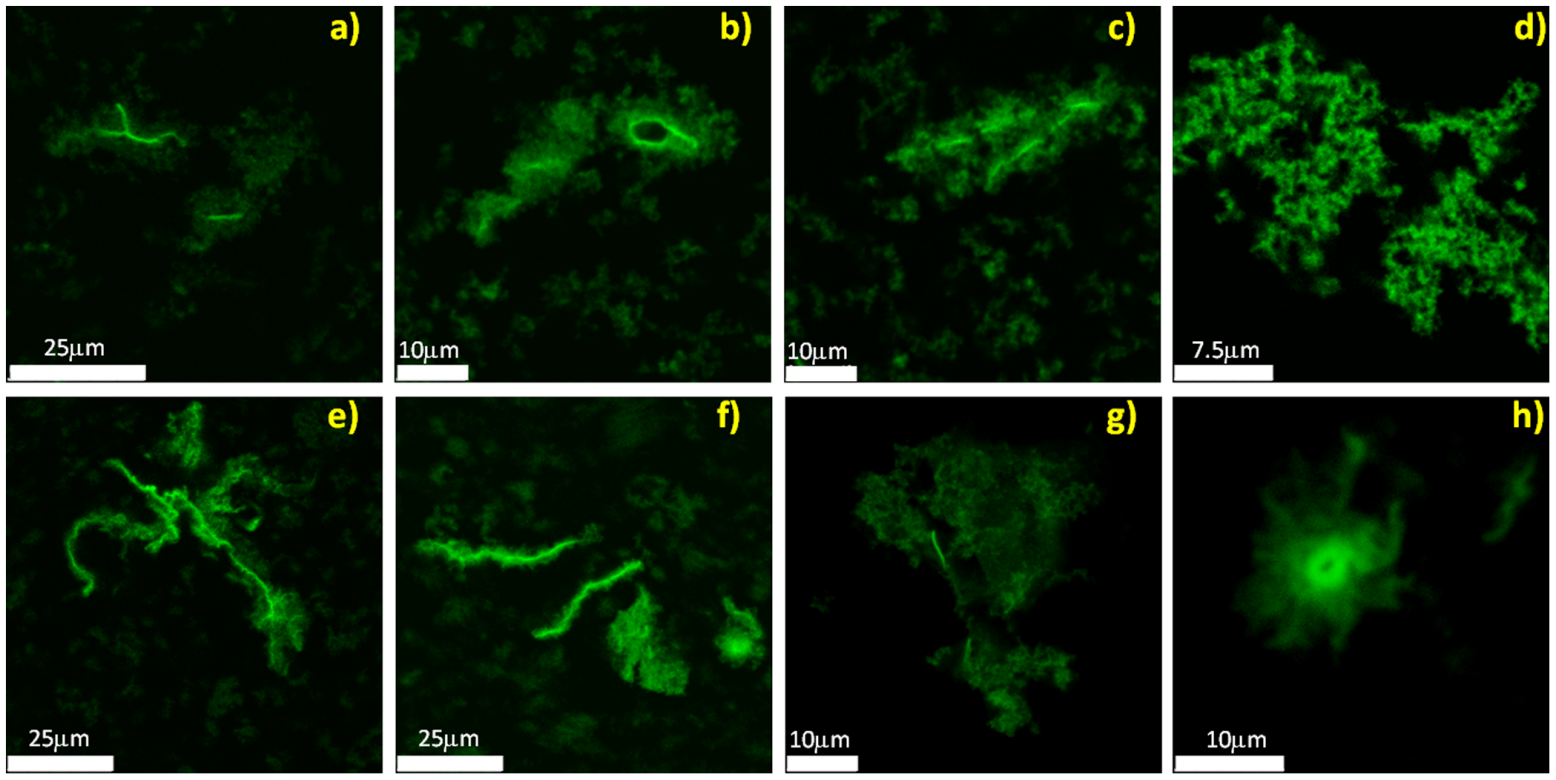
Morphologies of the occurring species. Two-photon excitation microscopy images for aggregates obtained from Con A sample at 20 µM and pH 3 after incubation at (a–d) 52°C (500 min) and (e–h) 62°C (200 min). Samples are stained by Thioflavin T.

Figures 3a and b present the evolution of the CD spectrum during the kinetics at both 52°C and 62°C for a 20 µM Con A sample. At room temperature Con A secondary structure is mainly β [17], this being quite independent of the pH of the solution. As can be seen in Figures 3a and b both at 52°C and 62°C the protein undergoes structural changes during the incubation, the changes at 62°C being more pronounced compared to the experiment at 52°C. Far-UV CD spectra progressively change their shape from a typical Con A β-native profile to a shape with a prominent peak at 215 nm that indicates the presence of β-aggregate structures. The ellipticity at 215 nm was used to follow the change in secondary structure during the process of amyloid formation (Figure 3c). At 52°C, the signal at 215 nm decreases without any further change after 300 min of incubation. A pronounced and steeper decrease of the signal is detected at 62°C and the signal reaches a plateau value in approximately 100 min. It is worth noting the pronounced difference in the absolute change of the CD signal at the two temperatures. The growth of a negative peak at 215 nm stems for the conversion of Con A β-native structure towards β-aggregates [28]. However, in this particular case, since the decrease of the signal at lower wavelengths (region around 190 nm) is also observed, the formation of random coil structures cannot be excluded *a priori* [43]. Data in Figure 3 strongly suggest that incubation at 62°C leads to profound structural changes of the native state while the supra-molecular aggregation takes place (Figure 1b). Similar changes are only observed to a minor extent at 52°C. Differences in Figure 3c coupled to data in Figures 1 and 2 suggest the potential presence of interconnected phases mainly dependent, at least in our experimental conditions, on temperature. As a consequence, monitoring the process at different temperature can allow singling out specific phases and in principle differences in the occurring species. To test if the aggregation process leads to different species or to the same species, only forming with different temperature-dependent rates, samples were stained using Thioflavin T dye and imaged by means of two-photon microscopy. Figure 4 shows representative images of the samples at the end of the kinetics both at 52°C (Figure 4 a–d) and 62°C (Figure 4 e–h). At lower temperature, clusters of small “crinkled” aggregates are observed. Specifically, they are formed by short elongated structures showing a low-intensity ThT signal and they are in the range of few µm. Together with such a structure, fewer large and brightly fluorescent fibrils are also present (Figure 4 a–c). At 62°C, the low fluorescent clusters are present but a sizeable amount of highly fluorescent fibrillar aggregates in the range of hundreds of µm in length can be detected. Such bright fibrillar structures are present in larger number compared to the few observed in the sample at 52°C and the higher ThT fluorescence intensity in such a fibrillar structures at 62°C stems for an increased amyloid-like nature and/or β-structure content. This matches with the larger secondary structure changes detected by CD for the sample at 62°C compared to the one at 52°C (Figure 3).

**Figure 5:**
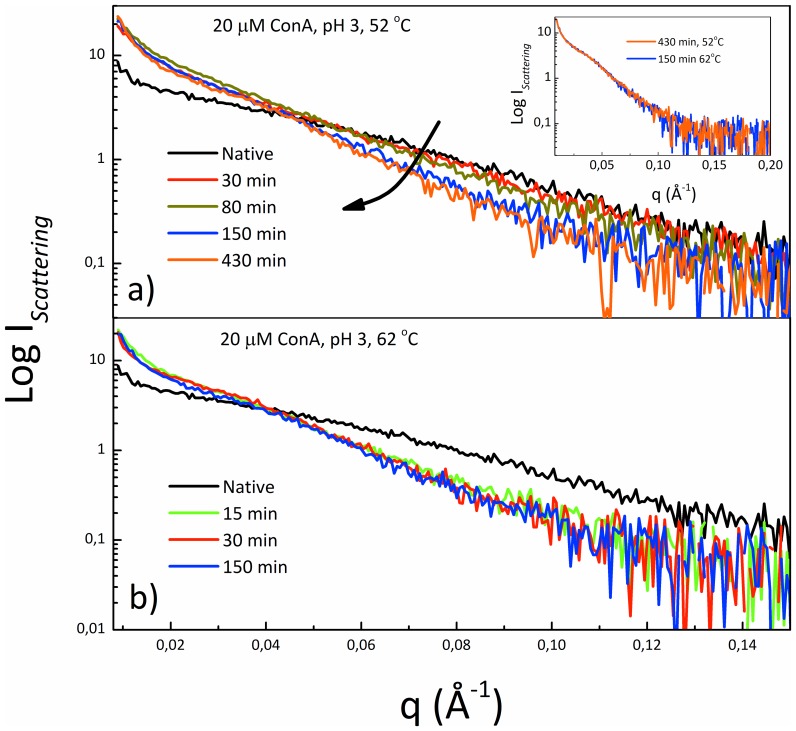
SAXS curves during the aggregation process. (a) SAXS signal detected at different time points of the aggregation kinetics of Con A sample at 20 µM and pH 3 during incubation at (a) 52°C and (b) 62°C. Inset: SAXS curves at the end of the kinetics at 52°C and 62°C, showing that they are superimposable.

Data in Figure 1, 2, 3 and 4 allow us to reach a first conclusion. The empiric fitting of Figure 1 suggests that the aggregation process follows the same overall pathway at all the temperatures (i.e. b = 1.4 for all the temperatures) but consists of interconnected and complex mechanisms/steps (i.e. compressed exponential fitting). Such mechanisms depend on temperature, as verified by the kinetics analysis in Figure 1 and Figure 2, and lead to aggregates with different secondary structures (Figure 3). Importantly, the kinetics of each phase is influenced differently by changes in the temperature, hence leading to different distributions of the analyzed final morphologies. That is, at 52°C the formation of “crinkled” aggregates (characterized by low ThT fluorescence intensity) is mainly observed with a slight presence of fibrils. On the contrary, at 62°C the process rapidly leads to the formation of large fibrillar structures (characterized by high ThT fluorescence intensity), even if the presence of crinkled aggregates can still be detected (Figure 4). Interestingly, for the sample in Figure 4 a–d, further incubation at 52°C up to 14 h results in an increase of the fibril number. Such images (not shown) resemble what is obtained in Figure 4 e–f for the sample incubated 200 min at 62°C. All together, the above mentioned evidences suggest at least the existence of two main phases/steps: an initial phase where the crinkled aggregates are formed and a second phase in which a further development and supra-molecular reorganization of such structures take place leading to the formation of fibrillar structures, resembling an aggregation-condensation mechanism. This brings us to conclude that the observed ThT-positive (low fluorescent) crinkled aggregates represent an on-pathway intermediate in the process of formation of amyloid-like elongated fibrils and their occurrence and stability are temperature-dependent. Such scenario is further confirmed by small-angle X-ray scattering (SAXS) analysis of the aggregation process. Figure 5 shows X-ray scattering data at different time-points for the kinetics at 52°C (Figure 5a) and 62°C (Figure 5b). At 52°C, SAXS curves show a specific development as a function of the time in the region 0.05<q<0.10 Å^−1^ before reaching the final state. This suggests the possibility to detect intermediate species during the kinetics. At 62°C, such development cannot be followed and only a transition from the native to the final state is detectable. Importantly, inset in Figure 5a clearly shows that identical scattering profiles are obtained for the final states at the two temperatures Data in Figure 5 confirm that the aggregation pathway is the same for the two conditions investigated (inset). However, using a specific temperature, i.e. 52°C, it is possible to detect the formation of long-lived intermediates preceding the formation of the final aggregated species (Figure 5a). As a consequence, differences between Figure 5a and 5b can again be explained by the difference in the temperature dependence of the two above mentioned aggregation-condensation phases. Deep analysis of the structural features contained in the SAXS data and a structural characterization of the on-pathway intermediate species are beyond the scope of the present work and will be subject of a future study.

### Tertiary Structural Changes and Role of Hydrophobic Interactions

In the previous section, the overall pathway of aggregation-condensation has been pointed out with temperature affecting the time scale of the two different steps. However, to further validate the above presented scenario, the microscopic mechanisms leading to such complex pathway need to be clarified.

**Figure 6:**
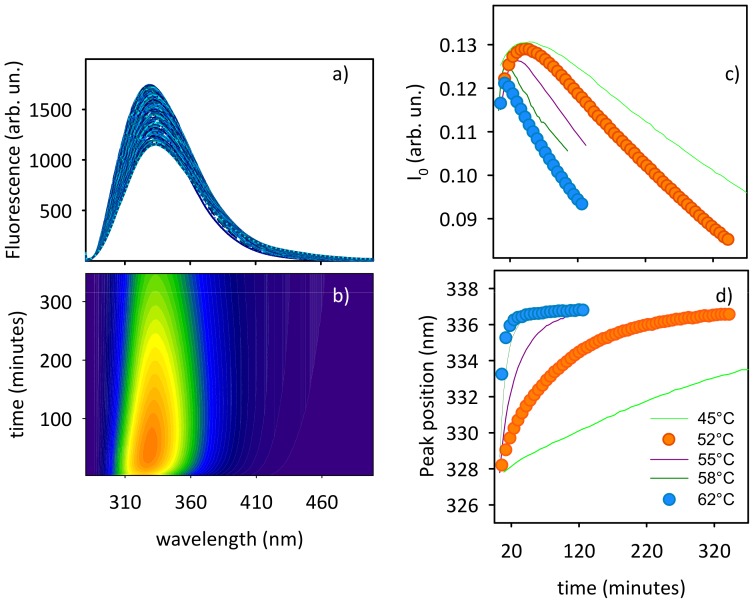
Tertiary structure changes during the aggregation process. (a) Tryptophanś bands during the aggregation of 20 µM Con A sample at pH 3 at 52°C and (b) contour plot with fluorescence increasing from blue to red. (c) Intensity of the Trp and (d) peak position as a function of time, for 20 µM Con A at pH 3 at different temperatures. Temperatures of interest, i.e. 52°C and 62°C, are indicated by circles.

The role of protein conformational changes in the observed supramolecular aggregation was followed by means of tryptophans (Trp) fluorescence. The intensity and the position of the intrinsic fluorescence band give valuable information on the conformational changes of Trp surroundings and polarity of their environment, respectively [23], [34], [44], [45]. In the case of Con A, Trp fluorescence arises from the four tryptophans located in different sites of each monomer and, for this reason, it only gives average information of the overall structural changes during the process. In Figure 6a we report the time evolution of Trp emission band for a sample of Con A 20 µM at pH 3 at 52°C. During the kinetics a red shift of the band is measured together with an increase of the fluorescence intensity followed by a slow decrease. Such a trend is clear in the contour plot (Figure 6b). To compare the Trp fluorescence changes at the two temperatures of interest (52 and 62°C), we analyzed the data in terms of spectral moments [see e.g. 23] obtaining the integrated fluorescence intensity (panel c) and mean peak position of the Trpś band (panel d) as a function of time. Fluorescence intensity shows biphasic behavior as a function of time, with an increase to a maximum value in the first phase while a decrease in the late phase of the kinetics is observed. We wish to note that a comparison between the absolute values of fluorescence intensity at the different temperatures is not possible due to the dependence of Trp quantum yield on temperature, but the comparison of the temporal profile of fluorescence signals during isothermal experiments gives important information on the occurring conformational changes and their time scale. The rate of both increase and decrease of fluorescence intensity in Figure 6c depends on temperature as also observed by further experiments (different temperatures within the 52–62°C range, solid lines in c). In Figure 6d a monotonic red shift of the mean peak position is observed as a function of time. The observed red shift appears to be temperature-dependent, i.e. being faster at higher temperature (solid lines in d) and the absolute change in position is approximately the same at each temperature. Like changes monitored by CD, the position of the Trp fluorescence band reaches a plateau in about 300 min and 100 min at 52°C and 62°C, respectively. Moreover, the temporal profile of the peak position at each temperature parallels with the aggregation kinetics monitored by light scattering (Figure 1a). On the contrary, data in Figure 6c do not show correlation with data in panel d but confirms a two-phase behavior.

Data in Figure 6 indicate a global rearrangement of protein tertiary structure in the local environment around the tryptophans of Con A, occurring in parallel with supra-molecular association. As reported above, the temporal evolution of the Trp signal takes into account the multiple environments around the four tryptophans. As a consequence, this rearrangement may either reflect changes in multiple environments within a single protein structure or arise from a mixture of more than one protein conformation coexisting in the sample. The reorganization of protein structure during supra-molecular assembly appears to be characterized by a complex pathway with two distinguishable main phases as monitored by the biphasic temporal evolution of Trp fluorescence intensity. At the beginning of the kinetics the increase of Trp quantum yield can be ascribed to changes in the local environment of these residues occurring in parallel with protein oligomerisation. The following quenching of the fluorescence can be due to the alignment or stacking of Trp that possibly occur during β-aggregates formation, with molecular interaction that may favor quenching mechanisms [46]. Moreover, the observed monotonic red shift of the fluorescent band reflects changes in the overall environment of Trps initially buried in native structure and then progressively exposed to the solvent during the aggregation pathway.

**Figure 7:**
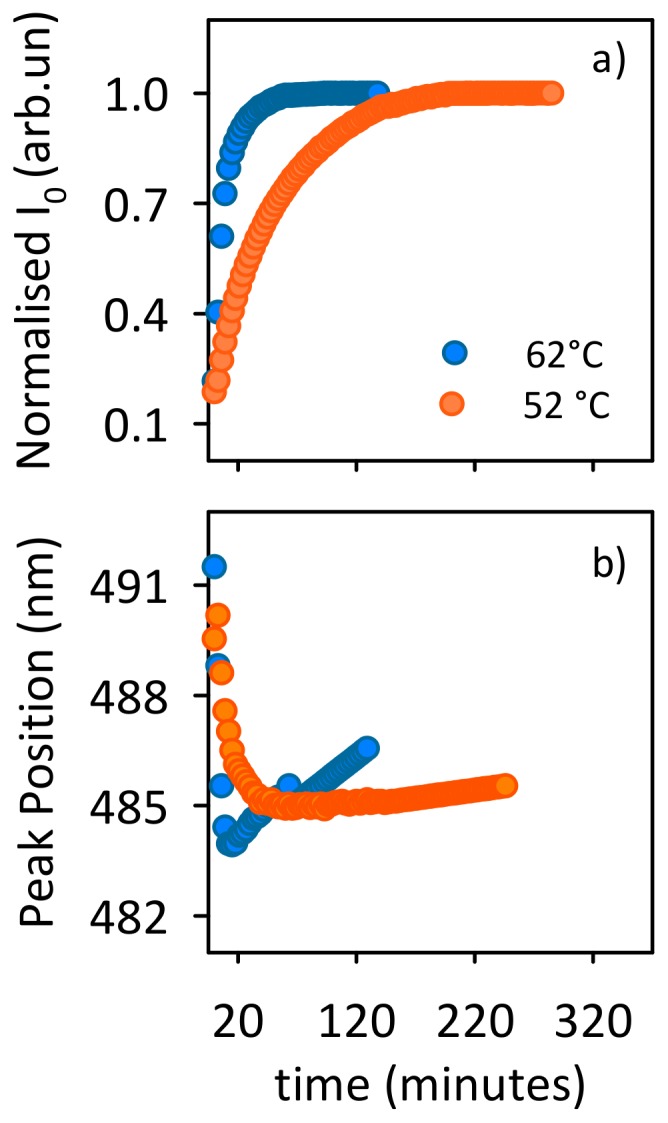
Changes in hydrophobic regions during the aggregation process. ANS fluorescence (a) intensity and (b) peak position as a function of time, for 20 µM Con A at pH 3 at 52°C and 62°C.

Such scenario is further confirmed by the analysis of ANS kinetics reported in Figure 7. ANS is widely used to monitor structural changes involving hydrophobic regions. These regions are either already present in native protein structures or formed during the aggregation process [23], [47]–[50]. The enhancement of ANS fluorescence intensity indicates an increase of exposed hydrophobic regions, which are able to bind ANS. It is important to note that this dye only slightly interacts with the native state of the protein [21]. Hence, the observed intensity increase can be ascribed to the enhanced accessibility of new hydrophobic regions of the protein or to the formation of new hydrophobic clusters during the aggregation route [23], [47]–[50].

The kinetics of ANS fluorescence intensity growth (Figure 7a) is on the same time-scale as the scattering increase at both temperatures and this indicates a close relation between the temporal evolution of changes in hydrophobic areas and the progress of aggregate formation. Importantly, the position of the ANS band shows a biphasic behavior. Specifically a blue shift in the initial part of the process and then a red shift is detected (Figure 7b). This indicates that during the aggregation pathway, although binding sites for ANS are progressively formed, the nature of the protein-dye interaction changes, being less favorable in the last part of the kinetics [47]–[50].

All the above mentioned observations suggest that the process is divided into two main steps. In the early phase, tryptophans initially buried into protein interiors are progressively exposed to more polar surroundings due to local rearrangements of their environment at high temperature (red shift in Figure 6d). This mechanism also leads to the increase in the Trp fluorescence intensity in the early phases of the process (Figure 6c). At the same time conformational changes in single Con A molecules provide new binding sites for ANS and in turn generate the increase in ANS emission (Figure 7a) and the initial blue shift of ANS fluorescence band (Figure 7b), i.e. aggregation phase. In the following phase, i.e. coagulation phase, Con A aggregates undergo progressive structural reorganization during supra-molecular assembly with the modification of previously formed hydrophobic binding sites or with the formation of new sites with minor affinity or different binding mode to ANS (red-shift in Figure 7b). This process probably determines the concurrent quenching of Trp fluorescence (Figure 6c). The latter can be attributed both to solvent exposure and to local molecular interactions with neighbor residues [46].

According also to the results of the previous section, the same overall scenario described here is likely to take place at both temperatures. Data in Figure 6 and 7 indeed confirm once more that the overall pathway is the same at all the temperatures with two main interconnected temperature-dependent steps (biphasic behavior in Figure 6c and d and 7b). This fully supports and completes the aggregation-condensation framework presented in the previous section.

**Figure 8:**
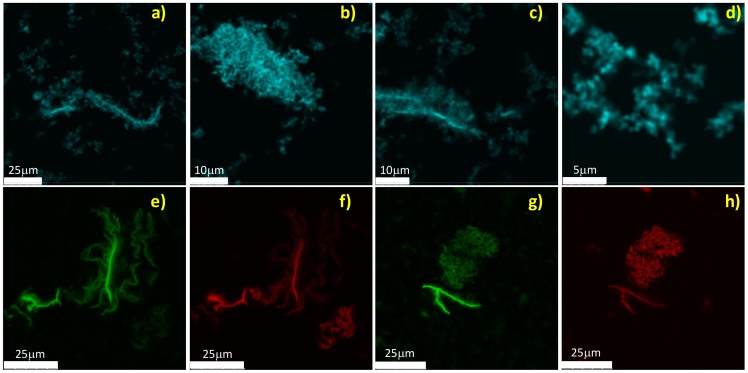
Hydrophobicity of the occurring species. Confocal and Two-photon excitation microscopy images for aggregates obtained from Con A sample at 20 µM and pH 3 after incubation at 62°C (200 min). Samples are stained by (a–d) ANS and (e–h) Hypericin in red and ThT in green (double color images).

Finally, to further investigate the nature of the observed structures, samples were stained with ANS dye (Figure 8a–d) and Hypericin, the last one in combination with ThT (Figure 8 e–h, see Materials and Methods for details) and imaged. Images are presented for samples at 62°C, a similar trend is observed for samples at 52°C except for the limited presence of fiber-like structures (not shown). ANS and Hypericin were used to observe hydrophobic regions of aggregates, independently on their fibrillar nature [51]. Data in Figure 8a–d confirm the hydrophobic nature of the observed structures. Both the clusters and the elongated structures present a bright ANS fluorescence. It is worth noting that the spine of the fibrillar structures is characterized by a fluorescence signal higher than the one from crinkled aggregates. This is corroborated by images in Figure 8 e–h where ThT and Hypericin show a higher fluorescence for fibrils while a low intensity is detected for the crinkled aggregates. Similar results are also observed in imaging experiments using other hydrophobic dyes as Sypro Orange and Nile Red (data not shown). These observations indicate a higher hydrophobic nature of the observed fibrillar structures.

### Conclusions

**Figure 9:**
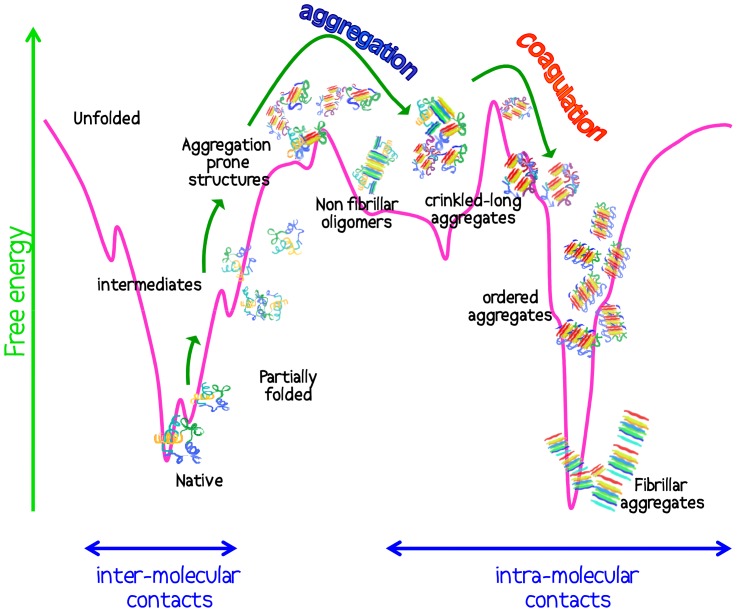
Representative scheme of the aggregation process. Con A aggregation mechanisms in the selected conditions: at high temperature partially folded destabilized states are accessible to protein molecule together with aggregation prone conformers which can form intra-molecular contacts leading to aggregates species and non-conventional fibrils which represent precursors of large fibrillar aggregates. Aggregation mechanisms appear to be regulated by two main energy barriers: at T∼52°C accessible states include non fibrillar oligomers, and long crinkled aggregates while at T∼62°C ordered oligomers and other aggregate species are accessible and they coagulate and form large fibrillar deposits.

This experimental work describes the aggregation kinetics of Con A at low pH. Previous studies at basic pH showed that the interplay of hydrophobic and electrostatics interactions far from the isoelectric point drives aggregation to the formation of long and thin fibrillar aggregates via a simple non-cooperative aggregation mechanisms [28], [29]. In the experimental conditions considered here, our data suggest that the flexibility of the initial Con A “unlocked” structure (dominant at low pH) and the varied charge state of the molecules lead to the observation of a significantly different phenomenon. The aggregation reaction proceeds through a non-nucleated supra-molecular assembly involving structural and conformational changes of the protein. The aggregation process proceeds via two phases and the two phases have different temperature dependency. Hence, at lower temperature (or shorter times at higher temperatures) crinkled structures (non–conventional fibrils) with low ThT fluorescence are observed as a result of initial conformational conversion and aggregation, while on a longer time scale (or earlier at higher temperatures) the coagulation of some of such on-pathway species in solution leads to the formation of more conventional β-rich fibrils extending up to hundreds of µm and with higher ThT emission. This picture is summarized in Figure 9. During the coagulation phase, intermolecular β-sheets in the “crinkled” precursor tend to coagulate into large fibrillar structures. We highlight that such a mechanism (with a higher energy barrier) depends, among other factors, on the strong interplay between hydrophobic and electrostatic forces. Such a scenario has been previously predicted by molecular simulation for generic polypeptide chains in terms of Ostwald step rule in first order phase transitions [52] and it has recently been experimentally observed for N47A mutant of the α-spectrin SH3 domain [53]. It is thus not highly controversial that an aggregation pathway could potentially pass through intermediate species, which later further condensate. What is however particular for the present study, is the observation that the two phases depend differently on a tunable external parameter (temperature). This allows us to single out the two phases of the process, and enables the further study of the intermediate quasi-stable species.

As a general result, our work suggests how the initial conformational flexibility of a protein can potentially influence the early stages and, consequently, the later events in the aggregation pathway. Our results, specifically obtained for Con A, may potentially be applicable to other fibrillating systems that undergo aggregation processes through well-defined energy barriers. It would hence become an instrumental tool to identify external parameters, which would potentially affect early and later aggregation phases differentially, helping in designing conditions in which long lived intermediate species can be opportunely isolated and their low-resolution structures and intrinsic cytotoxic properties can be elucidated. The above mentioned studies are currently in progress in our laboratories. This is particularly important also in the light of Con A capability in programmed-death of tumoral cells.

## Materials and Methods

### Sample Preparation

Concanavalin A (Con A, type IV, L7647), 8-Anilino-1-naphthalene-sulfonate (ANS), Hypericin and Thioflavin T (ThT) were purchased from Sigma Aldrich and used without further purification. All the measurements were performed in aqueous solution with 0.1 M KCl. pH was adjusted with HCl to a final value of 3. Each solution was freshly prepared and filtered just before the measurements through 0.22 µm filters (MS 16534, Sartorious). Con A aggregation was studied at different temperatures and protein concentrations. ANS and ThT were dissolved in the samples at a concentration of 25 µM and 36 µM, respectively. Concentration measurements were performed by means of a Shimadzu-240IPC UV-Vis spectrophotometer. Protein concentration was obtained using absorption at λ = 280 nm, and ε = 33 280 cm^−1 ^M^−1^. During aggregation kinetics, precipitation is observed for long time intervals, being temperature-dependent. To avoid artifacts due to this effect (both in the presence and in the absence of fluorescent dyes), absorption measurements were performed as a function of time in the range 190–650 nm. Aggregation kinetics reported in the paper is related to time interval in which sample absorption was constant, i.e. no precipitation.

### Dynamic Light Scattering

Scattering intensity during Con A aggregation was measured by dynamic light scattering employing a Zetasizer Nano ZS (Malvern Instruments) with a 633 nm light beam and operating in the back scattering configuration (173°). Experiments were performed at different temperatures, inducing protein aggregation on a sample volume of 1.1 mL in a PMMA UV-Grade cuvette (Kartell). Measurements were recorded every 3 or 5 min during the incubation. The activation energy was estimated considering the Arrhenius relation 1/τ_c_ = A exp (−E_a_/kT), being the 1/τ_c_ values obtained by the fitting of Figure 1b.

### Fluorescence Measurements

Fluorescence spectra were carried out on a Jasco FP-8500 equipped with peltier- thermostat and in a 1 cm path quartz cuvette. After thermal equilibration, the emission spectra were recorded with 0.5 nm wavelength intervals, emission and excitation bandwidth of 3 nm, scan-speed of 100 nm/min and integration time of 1 s. Spectra were analyzed by calculating the integrated intensity of the spectral distributions after subtracting the tangent to the minima of each band. The tryptophanś emission spectra were obtained under excitation at λ_exc_ = 275 nm in the emission range 280–430 nm. This wavelength is a good compromise between spectral quality and the requirement of avoiding significant tyrosine contributions to the emission. After thermal equilibration ANS emission spectra were acquired every 3 minutes under excitation at 380 nm, in the range 380–420 nm. Excitation spectra at 25°C, were measured after each kinetic measurement to check if any variation of the excitation band profile emerges. ThT emission spectra were detected using an excitation wavelength of λ_exc_ = 440 nm in the range 445–620 nm.

### Circular Dichroism

Measurements in the Far-UV region were carried out on a Jasco J-715 spectropolarimeter, equipped with a Jasco PCT 348 WI temperature controller. Sample cell paths were 0.2 mm for isothermal kinetics and 0.5 mm for the temperature scan, spectral resolution of 0.1 nm and averaging time of 1 s. Spectra were sequentially recorded, using a scan speed of 50 nm/min and a 1 nm bandwidth, data pitch of 0.1 nm. In order to follow the kinetics of the spectra, no average of scans was performed. CD and fluorescence measurements were made simultaneously, using different aliquots of the same freshly prepared sample. Variations in the absorption spectra and significant changes in the HT value during these measurements were not observed.

### Confocal and Two-Photon Fluorescence Microscopy

After aggregation, samples were stained with ANS, ThT and/or Hypericin (Sypro Orange and Nile Red were also tested). 10 µl aliquots of stained samples were placed on microscope slides and imaged at 1024×1024 pixel resolution using a Leica RCS SP5 confocal laser scanning microscope with a 63× oil objective NA = 1.4 (Leica Microsystems, Germany), a scanning frequency of 400 Hz. For imaging using ThT, the two-photon excitation (Spectra-Physics Mai-Tai Ti:Sa ultra-fast laser) was set at 885 nm and emission detected in the range 450–500 nm; for ANS experiments, excitation was set at 780 nm and emission detected in the range 430–530 nm. For Hypericin images, excitation was set at 543 nm (He-Neon laser) and the emission range was 620–720 nm. Pinhole was 95 µm. The spectroscopic properties of Hypericin allow simultaneously acquiring in a double-color experiment the signal of ThT (Figure 8 e–h) without significant bleed-through. In two color experiments images were sequentially acquired for ThT (green) and Hypericin (red) channel.

### SAXS Measurements

The SAXS experiments were performed at beamline I911-4 at MAXlab, Lund, Sweden, using a 61-period, 3.5 T, multipole-wiggler operating at a wavelength of 0.91 Å. Beam area is of 0.3×0.3 mm^2^. The scattering was registered on a Pilatus detector in the momentum transfer range of 0.008<q<0.45 Å^−1^. Sample exposure was 2 min. Buffers were measured before and after sample exposure, and averaged before background subtraction. Repeated exposure did not reveal any radiation damage. Samples at different time points of the kinetics were frozen and immediately measured after de-freezing them. The bioXTAS RAW [54] software was used for radial averaging and background subtraction. The average molecular mass of the protein was estimated from the extrapolated forward scattering I(0) by using a reference solution of bovine serum albumin. The molecular weight of the native state was ∼29±3 kDa with a radius of gyration of ∼ 5 nm. The radius of gyration (Rg) and the scattering intensity at zero angle I(0), for each sample was determined from the Guinier approximation using PRIMUS [55].
